# Preparation and Characterization of Ginseng Gel and In Vivo Evaluation of Its Clinical Efficacy in Generalized Chronic Gingivitis Patients

**DOI:** 10.7759/cureus.57097

**Published:** 2024-03-27

**Authors:** Johnisha Harris, Arvina Rajasekar

**Affiliations:** 1 Department of Periodontics, Saveetha Dental College and Hospitals, Saveetha Institute of Medical and Technical Sciences (SIMATS) Saveetha University, Chennai, IND

**Keywords:** chronic gingivitis, ginseng, chlorhexidine, anti-microbial, anaerobic microorganisms

## Abstract

Aim

The aim of the present study was to prepare and characterize ginseng gel and then to evaluate its clinical efficacy in terms of plaque index (PI), gingival index (GI) among generalized chronic gingivitis patients.

Materials and methods

Ginseng gel was prepared using 250 g of ginseng powder. The antimicrobial activity of prepared ginseng gel and chlorhexidine gel was checked at various concentrations (25, 50, 75, 100, 125, 150, 175, 200, 225, 250 and 275 µg) against anaerobic organisms to find the concentration with maximum antimicrobial activity. The concentration with highest antimicrobial activity was subjected to in vivo analysis. A total of 30 generalized chronic gingivitis patients were subjected to scaling and then divided into two groups for intraoral gel application - Group I (ginseng gel) and Group II (chlorhexidine gel) for one month. The clinical parameters PI, GI were measured at baseline (pre scaling) and one month (post scaling) comparing ginseng gel and chlorhexidine gel (Hexigel - chlorhexidine gluconate 1.0% w/w). Independent t test and paired t test were done for statistical analysis.

Results

At 275 µg, ginseng gel showed highest antibacterial action against anaerobic oral microorganisms. In Group I, the reduction in PI from baseline was (2.52±0.02) to follow up after one month (0.75±0.05), GI from baseline (2.2±0.35) to follow up after one month (0.9±0.02). In Group II, the reduction in PI from baseline was (2.54±0.01) to follow up after one month (0.79±0.02), GI from baseline (2.1±0.42) to follow up after one month (0.8±0.01).

Conclusion

Ginseng gel showed equal clinical efficacy to chlorhexidine gel in terms of PI and GI. Though chlorhexidine was effective in lower concentrations, it has considerable adverse effects such as taste alteration. Hence it is better to encourage the use of herbal-based products for the management of gingivitis to prevent side effects of synthetic preparations.

## Introduction

Periodontitis is a chronic inflammatory disease, primarily caused by periodontal pathogens both aerobic and anaerobic organisms which leads to the destruction of periodontal tissues. Dental plaque signifies the first association between periodontal pathogens and the oral cavity. A community of microorganisms adhered tenaciously to the tooth surface in the form of biofilm is called dental plaque biofilm [1]. The primary factor which influences the replication of microbes in a dental plaque biofilm is the extracellular polymers derived from the host, and bacteria. It has distinct characteristics that set it apart from similar organisms growing in liquid (planktonic) environments [2].

The primary etiology of periodontitis is facultative anaerobic bacteria like P. gingivalis and P. intermedia. Elimination of bacteria that harm periodontal tissues is one of the main goals for protecting against and preventing periodontal disease [3]. Plaque control can be done either by mechanical or chemical methods. Chemical agents that are used to control plaque include sanguinarine, Triclosan, CHX, CHX gluconate, and quaternary ammonium compounds. Gram-negative and positive bacteria are the primary targets of chlorhexidine (CHX), an agent that is effective against all microorganisms. The "pin-cushion Effect," which forms the foundation of the mechanism of action of chlorhexidine, can be explained as follows. According to this, while one end of the CHX molecule is attached to the tooth structure, the other end interacts with bacteria. The negatively charged phosphate and sulfate ions on the bacterial cell wall are attached to the bi-cationic positively charged molecule CHX. As a result, CHX is absorbed by the cell wall of the bacteria. The bacterial cell membrane is altered by CHX, and as the concentration of CHX increases, the cell membrane gradually becomes more damaged. When CHX attaches to the phospholipids in the inner membrane, low molecular weight substances like potassium ions leak out. Adenosine triphosphate and nucleic acid are two examples of phosphate complexes that cause coagulation and precipitation of cytoplasmic components, which ultimately results in bacterial cell death. Their large, rigid molecule with two charged ends gives an added advantage over other chemical agents. Being the strongest anti-plaque agent, chlorhexidine (CHX) is known as the "gold standard" anti-plaque agent. Its bacteriostatic and bactericidal qualities are attributed to its effectiveness, but not without side effects. Since chlorhexidine (CHX) is considered the gold standard for antibacterial agents, it has been extensively tested and used as a common positive control for assessing the antimicrobial activity of other drugs. Their application is to combat periodontal complications. On the other hand, prolonged, heavy use causes adverse effects. Teeth stains are one of the negative effects of chlorhexidine (CHX). It modifies the gustatory sensation of the patient as well. Thus, one should use it sparingly. As a result, the search for an antimicrobial agent that is equally effective with few to no adverse side effects is very essential. Determining the antibacterial activity of conventional plants is one feasible strategy [4].

Numerous plant extracts are used for their biological benefits all around the world. Their antimicrobial and anti-inflammatory properties might be the reason. Some are renowned for their anti-inflammatory, anti-oxidant, anti-bacterial and anti-fungal qualities [5-8]. The anti-inflammatory, antioxidative, and bacteriostatic qualities of ginseng and its preparations are well documented. Ginseng has demonstrated the ability to treat a wide range of infections. A previous study by Rokot et al., conducted on a broader population indicated that ginseng extract revealed potential therapeutic properties without any negative side effects, which is why it was widely employed in traditional Indian medicine [9]. In this context, the purpose of this study was to prepare and characterize ginseng gel and then to evaluate its clinical efficacy among generalized chronic gingivitis patients.

## Materials and methods

In vitro study

Preparation of Supercritical Fluid (SCF)

For 48 hours, 250 g of ginseng powder, which was obtained from dried ginseng leaves, was soaked in 1000 mL of ethanol. Using Whatman's filter paper, supercritical fluid (SCF) was filtered, evaporated and refrigerated.

Preparation of Ginseng Gel

**Figure 1:**
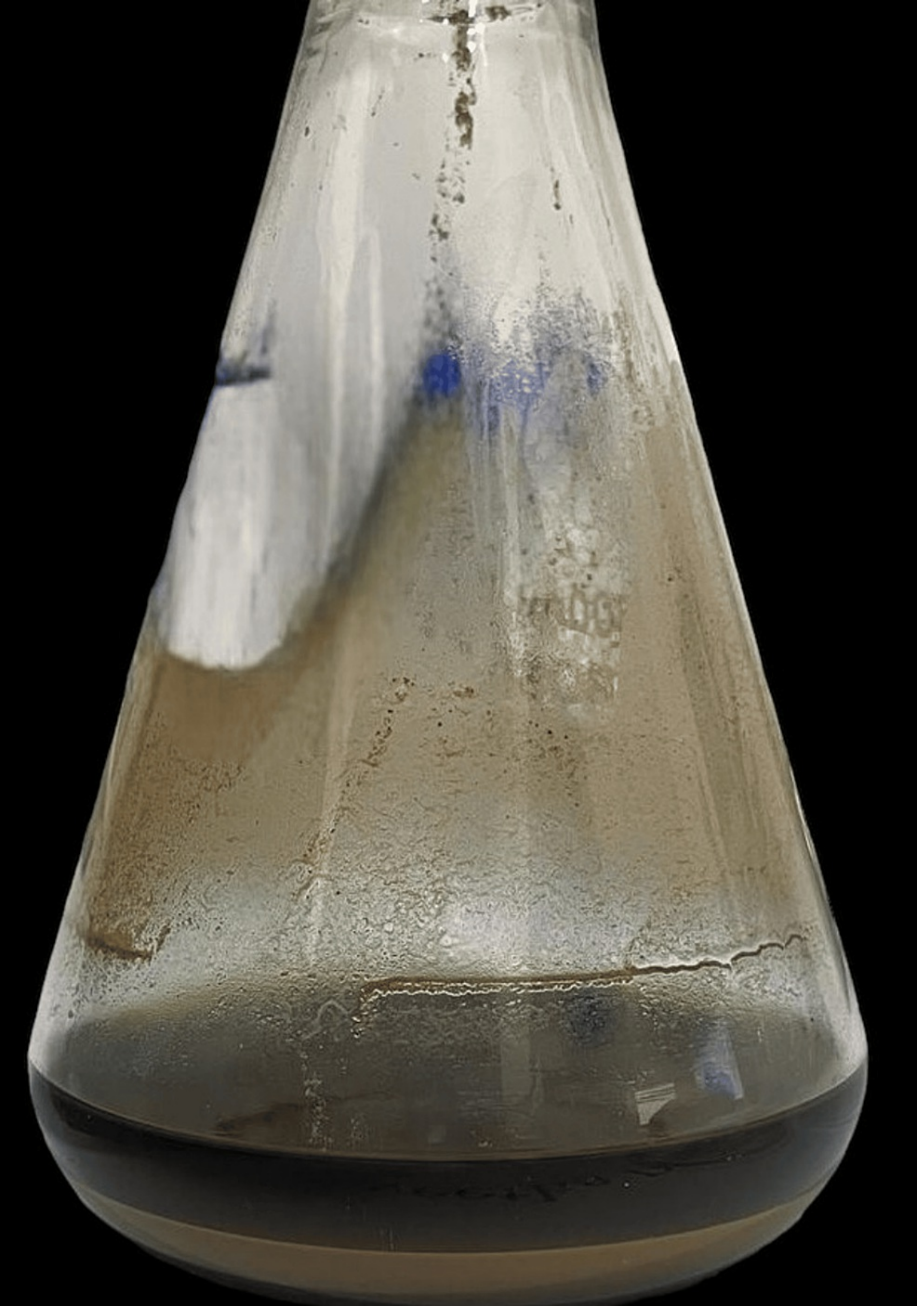
Extract containing 2 grams of Carbopol 940 and 0.2% sodium benzoate immersed overnight in distilled water.

**Figure 2:**
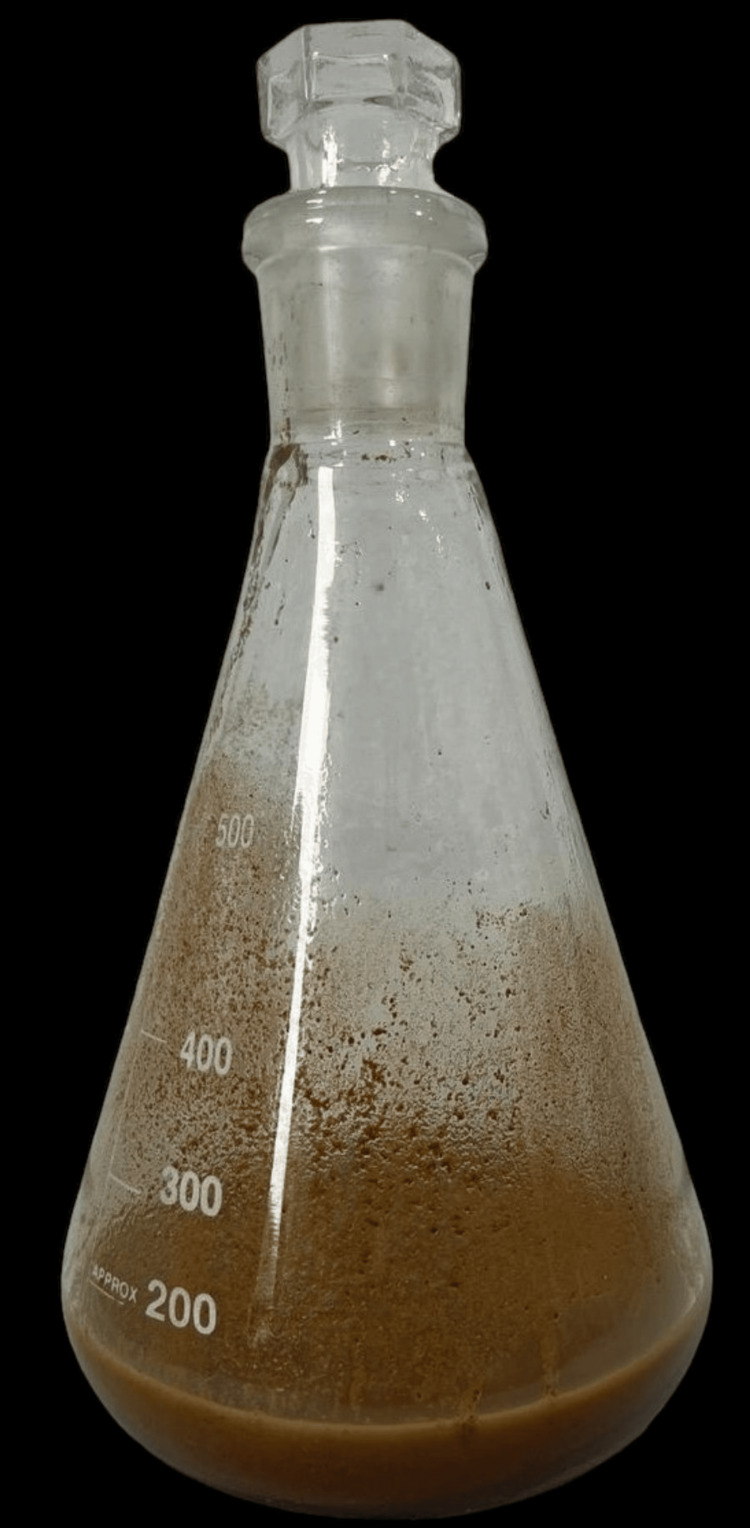
Final extract after the addition of ginseng homogenized supercritical fluid.

Two grams of Carbopol 940 was immersed overnight in distilled water containing 0.2% sodium benzoate which is shown in Figure 1. Two grams of hydroxypropyl methylcellulose (HPMC) solution and 5 ml of propylene glycol were added to it. Homogenized SCF (2 mL) was added to this mixture which gave a yellowish hue as shown in Figure 2. Drops of triethanolamine were added. The pH was adjusted to be between 6 and 6.5. The gel was stored at room temperature. For a period of six months, the consistency of the prepared gel was intact, similar to the time of preparation. Changes in pH were observed and corrected in accordance with the procedure.

Evaluation of Antimicrobial Property of Ginseng Gel

Subgingival plaque samples were taken from the deepest pocket of chronic periodontitis patients. After plaque collection with sterile Gracey curettes, the sample was quickly placed into an Eppendorf tube filled with thioglycollate broth. Thioglycollate in the medium absorbs oxygen and allows obligatory anaerobes to proliferate. The growth of the anaerobic microcolonies was encouraged by the use of anaerobic gas chambers containing anaerobic gas packs. The sample was incubated for 24 hours at 37 degrees in an anaerobic environment. This made it possible for the microbe in the soup to proliferate. One milliliter of distilled water was used to dilute the gels (test and control) into distinct concentrations (25, 50, 75, 100, 125, 150, 175, 200, 225, 225 and 275 µg). Twenty-four hours later, 20 µl of the cultured broth was subcultured into the test group (ginseng gel) and control group gels (chlorhexidine gel). Using an L-rod, 20 ml of each sample was distributed into the solidified BHI agar medium in a Petri dish. The Petri dishes were then incubated for 24 hours at 37 degrees in an anaerobic environment. To establish an anaerobic environment, anaerobic gas packs were put inside an anaerobic jar. The number of colonies that had formed after a day was counted using direct microscopy and recorded as colony-forming units (CFU).

In vivo study

Study Setting

This prospective clinical study was conducted among 30 outpatients diagnosed with generalized chronic gingivitis (AAP 2017 classification of periodontal diseases) at the Department of Periodontics, Saveetha Dental College and Hospitals. The study was done after approval by the Institutional Ethical Committee at Saveetha Dental College and Hospitals (IHEC/SDC/PERIO-2103/23/012). Sample size calculation was done using G*Power software, version 3.0, using the mean and standard deviation values from previous research [10]. Patients under the age group of 20-65 years who were systemically healthy were included in the study. Written patient consents were taken from all the participants. Periodontitis patients and those treated within the last six months, smokers, pregnant and lactating women were excluded from the study. All the subjects were subjected to full-mouth ultrasonic scaling. The subjects were then asked to massage the gingiva with either Ginseng gel (group I) or Chlorhexidine gel (Hexigel - chlorhexidine gluconate 1.0% w/w) (group II) two times a day for one month.

Study Group

GROUP I: Ginseng gel (Test group) (n=15)

GROUP II: Chlorhexidine gel (control group) (n=15)

The clinical parameters such as Plaque Index (PI) score and Gingival Index (GI) score were measured pre-scaling (baseline) and post-scaling along with Ginseng gel and Chlorhexidine (CHX) gel application in group I and group II respectively for one month. The clinical parameters PI and GI were recorded using the Silness and Loe plaque index (1964) and Loe and Silness gingival index (1963) respectively [11]. Clinical parameter assessment was carried out by a single examiner at baseline and one month after scaling.

Statistical analysis

SPSS Software version 23.0 (IBM Corp., Armonk, NY, USA) was used to analyse the data. PI and GI scores at baseline and at one month between study groups were compared using an independent t-test. PI and GI scores between different time periods in both study groups were compared using paired t-tests.

## Results

**Figure 3:**
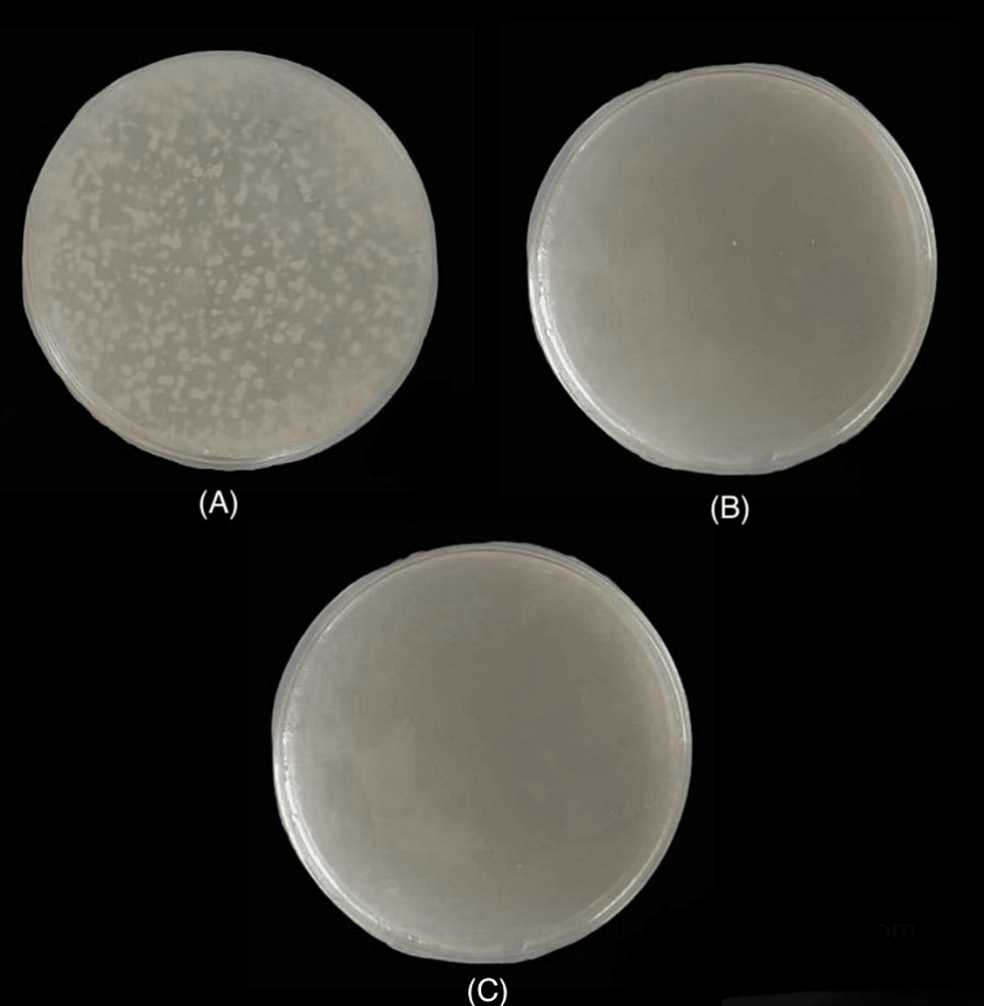
(A) 300 colonies in the test group at a concentration of 25 µg. (B) Three colonies in the test group at a concentration of 275 µg. (C) No colonies in the control group at a concentration of 25 µg.

The total number of colony-forming units observed at different concentrations (25, 50, 75, 100, 125, 150, 175, 200, 225, 250 and 275 µg) of ginseng gel and chlorhexidine gel were illustrated in Table 1. Figure 3 shows 300 colonies in the test group at a concentration of 25 µg and 3 colonies in the test group at a concentration of 275 µg and no colonies in the control group at a concentration of 25 µg respectively.

**Table 1:** Represents the number of colony-forming units (CFU) while assessing the antimicrobial activity at different concentrations of ginseng gel and chlorhexidine gel.

Concentration	Ginseng gel (CFU)	Chlorhexidine gel (CFU)
25 μg	300	0
50 μg	250	0
75 μg	225	0
100 μg	200	0
125 μg	175	0
150 μg	120	0
175 μg	75	0
200 μg	25	0
225 μg	15	0
250 μg	10	0
275 μg	3	0

In Group I, the reduction in PI from baseline was (2.52±0.02) to follow up after one month (0.75±0.05), GI from baseline (2.2±0.35) to follow up after one month (0.9±0.02). In Group II, the reduction in PI from baseline was (2.54±0.01) to follow up after one month (0.79±0.02), GI from baseline (2.1±0.42) to follow up after one month (0.8±0.01). The intergroup comparison of clinical parameters PI and GI between both the groups using an independent t-test is depicted in Table 2. On intergroup comparison of the post-operative clinical parameters between both the groups, there was no statistically significant difference between the two groups in terms of PI and GI (p > 0.05) (Table 2).

**Table 2:** Comparison of clinical parameters between two groups by independent t-test.

Parameters	Timeline	Group 1 (Mean±SD)	Group 2 (Mean±SD)	p-value
Plaque index (PI)	Baseline	2.52±0.02	2.54±0.01	0.25
	1 month	0.75±0.05	0.79±0.02	0.35
Gingival index (GI)	Baseline	2.2±0.35	2.1±0.42	0.39
	1 month	0.9±0.02	0.8±0.01	0.42

The intragroup comparison of clinical parameters PI and GI between pre-ginseng (baseline) and post-ginseng (one month), as well as pre-CHX (baseline) and post-CHX (one month) using paired t-test, is depicted in Table 3. An intragroup comparison of the clinical parameters within the groups showed a statistically significant difference (p < 0.05) in terms of PI and GI in both groups I and II (Table 3).

**Table 3:** Comparison of mean clinical parameters between baseline and one month in group 1 and group 2 by paired t-test. * statistically significant

Parameters	Timeline	Group 1 (Mean±SD)	p-value	Group 2 (Mean±SD)	p-value
Plaque index (PI)	Baseline	2.52±0.02	0.02*	2.54±0.01	0.02*
	1 month	0.75±0.05		0.79±0.02	
Gingival index (GI)	Baseline	2.2±0.35	0.03*	2.1±0.42	0.01*
	1 month	0.9±0.02		0.8±0.01	

The patient-based outcomes followed by the usage of ginseng gel and CHX gel are depicted in Table 4. None of the patients of both the groups reported discomfort, burning sensation, dryness/soreness or staining of teeth. However, taste alteration was reported by four patients of group II (chlorhexidine gel) (Table 4).

**Table 4:** Patient-based outcomes to analyze any adverse effects. CHX - Chlorhexidine

Parameters	Yes (n)		No (n)	
	Ginseng	CHX	Ginseng	CHX
Discomfort	0	0	0	0
Burning Sensation	0	0	0	0
Dryness/Soreness	0	0	0	0
Taste alteration	0	4	0	0
Staining of teeth	0	0	0	0

## Discussion

Phytotherapy has been a part of the medical field in treating various diseases since 2000 B.C. Since ancient times, plant extracts have been formulated from barks, seeds, fruits, stems, etc. It has been demonstrated that the phytochemicals in the herbs help to inhibit the growth of oral microbes [12]. The chief phytochemical components such as tannins, flavonoids, alkaloids, and quinones play a critical role in the anti-bacterial, anti-microbial and wound-repairing properties. Additionally, they reduce their ability to replicate in host tissues. This has led to the use of herbal-based products such as neem, curcumin, tulsi, etc. in dentistry over the last few years especially due to factors such as fewer side effects, safe and environment friendly [13].

Ginseng is one of those herbs known for its anti-inflammatory, anti-bacterial and antioxidant properties, combined with its lower toxicity. It has demonstrated potential benefits in the treatment of periodontal disorders [14]. Hence the present study aims to assess ginseng extract's antibacterial potential against anaerobic oral microorganisms. A cytotoxicity test revealed that ginseng gel was extremely biocompatible and less harmful than antimicrobials. In an in vitro study, ginseng gel showed strong antioxidant and anti-inflammatory properties on par with conventional medication. Due to its antimicrobial properties, it is frequently employed in the treatment of a number of systemic illnesses [15]. In our investigation, ginseng gel showed significant antibacterial activity against anaerobic oral microorganisms at a concentration of 275 µg. However, significant antibacterial activity was observed even at the lowest concentration of CHX gel. Furthermore, in our clinical study, it was observed that there was a statistically significant difference in PI and GI score in group I (ginseng gel) and group II (CHX gel) at one-month follow-up from baseline. The results of the present study cannot be directly compared with that of any other study because, to the best of our knowledge, it is one of the first studies of its kind, evaluating the effectiveness of ginseng gel as a local drug delivery agent in terms of clinical parameters in gingivitis patients. Therefore, the findings in the present study are indirectly compared with the previous studies, where other herbal gels have been used as an adjunct to scaling in gingivitis patients in terms of clinical parameters.

In the study by Kandwal et al., turmeric gel was compared against chlorhexidine gluconate which showed a significant difference in the plaque and gingival index which coincides with our results [16]. Another randomized control trial conducted by Ambhore and Padhye compared Triphala gel against CHX gluconate gel and checked for plaque, gingival and sulcular bleeding index. The results revealed a significant difference between baseline and one month with no significant difference between groups [17]. Similar results were seen in a study that compared curcumin gel against CHX gel with significant improvement in the clinical parameters after gel application in both groups [18]. These studies showed that herbal drugs when used in the optimum concentration yielded results similar to that of chemical drugs.

Limitations and future direction

The antibacterial activity of ginseng gel was not studied against specific periodontal pathogens. Also, the present study was done among smaller samples with shorter follow-up. Therefore, further randomized controlled clinical trials with larger sample size and long-term follow-up are needed to substantiate the findings of the present study.

## Conclusions

Ginseng gel showed equal clinical efficacy to chlorhexidine gel in terms of PI and GI. The efficacy increased with an increase in the concentration of ginseng, when the parameters PI and GI were compared between baseline and at one month. Though chlorhexidine was effective in lower concentrations, it has considerable adverse effects such as taste alteration. Ginseng has proven to be better in terms of patient outcomes without any adverse effects like chlorhexidine. Hence, it is better to encourage exploring the use of herbal-based products as a potent therapeutic agent for the treatment of gingivitis in the future.
